# Stress Concentration Induced by the Crystal Orientation in the Transient-Liquid-Phase Bonded Joint of Single-Crystalline Ni_3_Al

**DOI:** 10.3390/ma12172765

**Published:** 2019-08-28

**Authors:** Hongbo Qin, Tianfeng Kuang, Qi Li, Xiong Yue, Haitao Gao, Fengmei Liu, Yaoyong Yi

**Affiliations:** 1 Guangdong Provincial Key Laboratory of Advanced Welding Technology, Guangdong Welding Institute (China-Ukraine E.O. Paton Institute of Welding), Guangzhou 541630, China; 2 Key Laboratory of Guangxi Manufacturing System and Advanced Manufacturing Technology, Guilin University of Electronic Technology, Guilin 541004, China

**Keywords:** Ni_3_Al, transient liquid phase bonding, stress concentration, crystal orientation, finite element analysis

## Abstract

Single-crystalline Ni_3_Al-based superalloys have been widely used in aviation, aerospace, and military fields because of their excellent mechanical properties, especially at extremely high temperatures. Usually, single-crystalline Ni_3_Al-based superalloys are welded together by a Ni_3_Al-based polycrystalline alloy via transient liquid phase (TLP) bonding. In this study, the elastic constants of single-crystalline Ni_3_Al were calculated via density functional theory (DFT) and the elastic modulus, shear modulus, and Poisson’s ratio of the polycrystalline Ni_3_Al were evaluated by the Voigt–Reuss approximation method. The results are in good agreement with previously reported experimental values. Based on the calculated mechanical properties of single-crystalline and polycrystalline Ni_3_Al, three-dimensional finite element analysis (FEA) was used to characterize the mechanical behavior of the TLP bonded joint of single-crystalline Ni_3_Al. The simulation results reveal obvious stress concentration in the joint because of the different states of crystal orientation between single crystals and polycrystals, which may induce failure in the polycrystalline Ni_3_Al and weaken the mechanical strength of the TLP bonded joint. Furthermore, results also show that the decrease in the elastic modulus of the intermediate layer (i.e., polycrystalline Ni_3_Al) can relieve the stress concentration and improve the mechanical strength in the TLP bonded joint.

## 1. Introduction

Owing to their extremely high tensile strength, toughness, endurance strength, fatigue strength, corrosion resistance, and oxidation resistance at high temperatures (such as ≥980 °C), nickel(Ni)-based superalloys have been widely applied in aviation, aerospace, and military fields [1,2,3]. Because of the poor casting performance of Ni-based superalloys and the complexity of components, Ni-based superalloys usually need to be welded for their applications. The shortcomings of fusion welding and brazing in the connection of Ni-based superalloys (such as shrinkage stresses induced by rapid precipitation, formation of brittle phases, and the tendency to crack due to local fragility, etc.) limit the industrial application of the process for Ni-based superalloys [4,5]. Paulonis et al. proposed a transient liquid phase (TLP) bonding method [4], where in a thin layer of intermediate alloy with a lower melting temperature is employed as the connecting material. During TLP bonding, a low-melting liquid phase is formed between the parent material and the intermediate layer by heating under vacuum conditions; the liquid phase is then homogeneously diffused and therefore isothermally solidified, finally forming a joint with a uniform microstructure. Owing to its reasonable welding temperature, low applied pressure requirement, and ability to form a welded joint with excellent mechanical properties, TLP bonding is widely considered as one of the most ideal welding methods for Ni-based superalloys [5,6,7].

Grain boundaries, as surface imperfections, adversely affect the high-temperature mechanical properties of metals, especially their high-temperature endurance strength. Thus, the high-temperature mechanical properties of single-crystalline alloys are substantially better than those of polycrystalline alloys. Accordingly, single-crystalline alloys are extensively used as the latest generation of Ni-based superalloys. In the TLP bonding of homogenous Ni-based single-crystalline alloys, because the intermediate-layer alloy is a foil-belt amorphous or powdery Ni-based alloy, a region of polycrystalline Ni-based alloy is unavoidably formed in the joint after TLP bonding; the single-crystalline Ni-based alloy (i.e., parent alloy) is usually connected by a polycrystalline Ni-based alloy (i.e., intermediate layer) after TLP bonding. Extensive investigations on the compositions, heat treatments, mechanical properties, and simulations of single-crystalline Ni-based alloys have been reported [8,9,10,11,12,13,14,15]. In addition, some experimental studies that focus on the bonding process, intermediate-layer alloys, or joint microstructures in the welding joints of single-crystalline Ni-based alloys have recently been published [7,16,17,18,19,20]. Thus far, because of limitations in experimental characterization methods (e.g., the magnitude and distribution of stress and strain in the TLP bonded joint cannot be experimentally measured), few studies have been conducted to analyze the influence of the different states of crystallographic orientations between the single-crystalline parent material and the polycrystalline intermediate layer on the mechanical behavior of a TLP bonded joint.

Ni_3_Al-based alloys belong to Ni-based superalloys, which have excellent mechanical and physical properties [21]. In the present study, the mechanical properties of single-crystalline and polycrystalline Ni_3_Al were calculated by first-principles calculations based on density functional theory (DFT). In addition, the mechanical behavior of the TLP bonded joint of single-crystalline Ni_3_Al under a simple tension load was studied via the finite element (FE) method, and the stress concentration caused by the different states of crystallographic orientations between the single-crystalline parent alloy and the polycrystalline intermediate layer was evaluated.

## 2. Simulation Methods and Details

**Figure 1:**
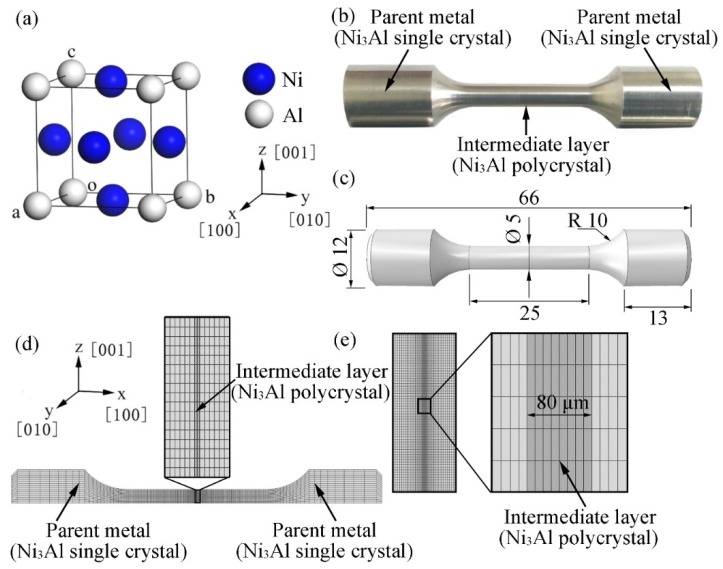
TLP bonded joint sample: (**a**) crystal structure of cubic Ni_3_Al, (**b**) a sample prepared in experiments, (**c**) the geometry of the sample for FE modeling (mm), and (**d**) the one-fourth 3D FE model and (**e**) submodel.

Intermetallic compound Ni_3_Al has a face-centered cubic lattice structure and *Pm*-3*m* space group; its lattice constant is *a* = 3.572 Å [22], as shown in Figure 1a. In this study, the CASTEP program [23] was used to perform first-principles calculations based on DFT. In the lattice-structure optimization and elastic-constant calculation, the local density approximation proposed by Ceperley and Alder was applied to investigate the exchange-correlation potential [24]. In addition, Vanderbilt ultra-soft pseudo potentials [25] and the Broyden–Fletcher–Goldfarb–Shanno algorithm [26] were also used during the lattice-structure optimization. The energy cutoff was taken as 600 eV, the *k*-points were set to 10 × 10 × 10, and the convergence tolerance of energy was set as 5.0 × 10^−6^ eV/atom. The self-consistent field had a convergence accuracy of 5.0 × 10^−7^ eV/atom, and the maximum ionic Hellmann–Feynman force was 0.01 eV/Å. The stress deviation during the calculation was less than 0.02 GPa.

The length of the TLP bonding sample, the diameter of the joint, and the thickness of the intermediate layer were 66 mm, 5 mm, and 80 μm, respectively, as shown in Figure 1b,c.

A simple tension load of 50 MPa was applied to the ends of the sample at room temperature. Given the axisymmetry of the sample, a one-fourth (1/4) symmetric FE model was established for three-dimensional (3D) finite element analysis (FEA) (Figure 1d), which can drastically reduce calculations and save time. The FE model comprised the parent alloy (i.e., single-crystalline Ni_3_Al) and an intermediate-layer alloy (i.e., polycrystalline Ni_3_Al), and there were 50,688 elements and 221,201 nodes. Obviously, the mesh density of the TLP bonded joint in the model, as further shown in the magnification of the indicated zone in Figure 1d, was not sufficiently fine to accurately analyze the localized stress and strain distribution; thus, the submodel method was adopted in the FEA on the basis of Saint-Venant’s principle, as displayed in Figure 1e. The location of the submodel is the same as the indicated zone in Figure 1d, and the submodel comprises 186,850 elements and 775,736 nodes. All FE calculations were performed with the ABAQUS 2018 software, and quadratic complete integration (C3D20) and full Newton iteration were employed to accurately solve the stress–strain relationship.

## 3. Results and Discussion

### 3.1. Mechanical Properties of Ni_3_Al

In the elastic stage, the stress and strain relationship can be described by Hooke’s law: *σ_ij_* = *D_ijkl_ε_ij_*, where *D_ijkl_* denotes the elastic constants. According to the symmetry of the crystal lattice, the elastic stress–strain matrix **[*D_ijkl_*]** of single-crystalline Ni_3_Al can be expressed as

$$\left(\begin{array}{c} \sigma_{11}\\ \sigma_{22}\\ \sigma_{33}\\ \sigma_{12}\\ \sigma_{13}\\ \sigma_{23} \end{array}\right)=\left(\begin{array}{cccccc} D_{1111} & D_{1122} & D_{1133} & 0 & 0 & 0\\ & D_{2222} & D_{2233} & 0 & 0 & 0\\ & & D_{3333} & 0 & 0 & 0\\ & & & D_{1212} & 0 & 0\\ & Sym. & & & D_{1313} & 0\\ & & & & & D_{2323} \end{array}\right)\left(\begin{array}{c} \varepsilon_{11}\\ \varepsilon_{22}\\ \varepsilon_{33}\\ \gamma_{12}\\ \gamma_{13}\\ \gamma_{23} \end{array}\right)$$

where *σ_ij_*, *ε_ij_*, and *γ_ij_* are the stresses, normal strains, and shearing strains respectively. The stiffness matrix **[*D_ijkl_*]** was calculated by linearly fitting four small strains (±0.001 and ±0.003) under nine deformation conditions (see Supplementary Figure S1); the calculated elastic constants are listed in Table 1. The flexibility matrix **[*S_ijkl_*]** was calculated as the inverse matrix of the stiffness matrix **[*D_ijkl_*]**, i.e., **[*S_ijkl_*] = [*D_ijkl_*]**^−1^; the results for elastic constants *S_ijkl_* are given in Table 2.

According to the Voigt–Reuss–Hill approximation method [28], the bulk modulus (*B*) and shear modulus (*G*) of polycrystalline Ni_3_Al can be calculated by Equations (1) and (2), respectively:

(1) $$B=\frac{1}{2}\left[\frac{1}{3S_{1111}+6S_{1122}}+\frac{1}{3}\left(D_{1111}+2D_{1122}\right)\right],$$

(2) $$G=\frac{1}{2}\left[\frac{15}{4S_{1111}-4S_{1122}+3S_{1212}}+\frac{1}{5}\left(D_{1111}-D_{1122}+3D_{1212}\right)\right].$$

The calculated values of *B* and *G* are 186.72 GPa and 78.86 GPa respectively. Moreover, the elastic modulus (Young’s modulus, *E*) and Poisson’s ratio (*v*) can be evaluated by Equations (3) and (4) respectively:

(3) $$E=\frac{9BG}{3B+G},$$

(4) $$\nu =\frac{3B-E}{6B}.$$

The calculated values of *E* and *v* for polycrystalline Ni_3_Al are 207.38 GPa and 0.315 respectively; these values are similar to the experimental values *E* = 203.1 GPa and *v* = 0.305 at room temperature [29].

### 3.2. Stress Concentration Induced by the State of Crystal Orientation

**Figure 2:**
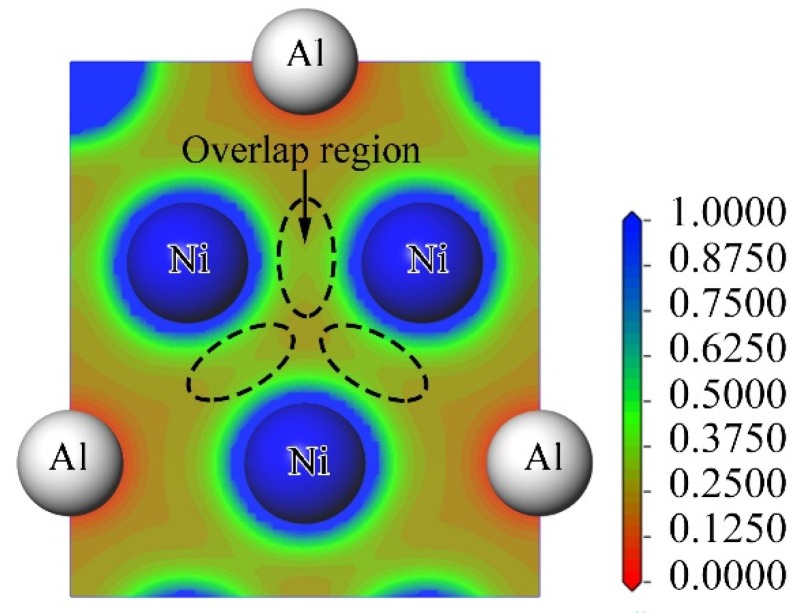
Distribution of electron density in the (111) crystal plane.

The calculated elastic constants *D_ijkl_* and the aforementioned mechanical properties were used in FEA for a tension load of 50 MPa, which is much less than the yield strength of Ni_3_Al (>350 MPa [30]). Covalent bonding and ionic bonding are well known to lead to brittleness in intermetallic compounds. Herein, the crystal plane (111) was selected to evaluate the chemical bonds between Ni–Ni and Ni–Al atoms, and the electron density in the (111) planes was calculated via DFT (Figure 2). From the overlap of the electron densities of Ni–Ni atoms, it is inferred that covalent bonds exist. Meanwhile, the electron charge density around Ni atoms is 1.0, whereas that close to Al atoms is 0.0, indicating that the electrons of Al atoms move toward the Ni atoms and that Ni and Al atoms are bonded together by ionic bonds. Therefore, we assumed that Ni_3_Al exhibits some brittleness.

**Figure 3:**
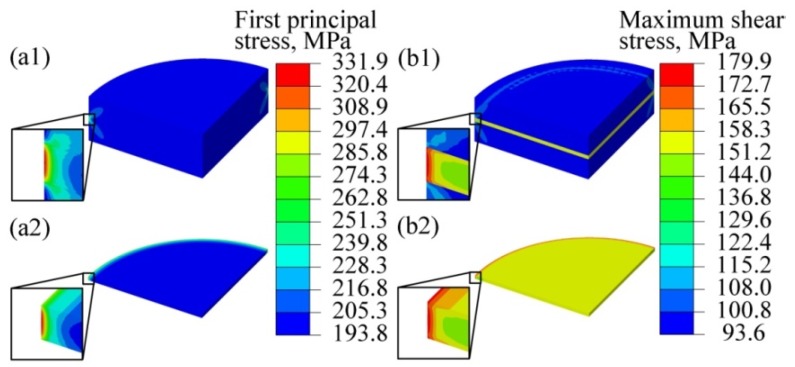
Simulation results of first principal stresses and shear stress: first principal stress in (**a1**) the TLP bonded joint and (**a2**) the intermediate layer; and shear stress in (**b1**) the TLP bonded joint and (**b2**) the intermediate layer.

According to the maximum principal stress theory, in a simple tension test, failure occurs if the first principal stress (*σ*_1_) reaches the elastic limit stress. Figure 3a1,a2 display the distribution of *σ*_1_ in the submodel and the intermediate layer, where the *σ*_1_ is maximally localized at the edge of the intermediate layer (refer to the red contour). The maximum *σ*_1_ and average *σ*_1_ are 331.9 and 200.1 MPa respectively, and the ratio of the maximum *σ*_1_ to average *σ*_1_ is 1.66. Clearly, different states of crystal orientation induce stress concentration in the intermediate layer, which may lead to failure in this layer. Moreover, Pugh et al. [31] proposed that the ratio between the bulk modulus and the shear modulus (i.e., *B*/*E*) can be used to evaluate the ductility of a material; they also proposed that ductile materials have *B*/*E* values greater than 1.75. The calculated *B*/*G* ratio for Ni_3_Al in this study is 2.37; we therefore speculate that Ni_3_Al also has some ductility, which can also be verified from the experimental results reported elsewhere [32].

The maximum shear stress theory assumes that failure occurs when the maximum shear stress reaches the yield point measured in the simple tension test, which is often used during the strength design of ductile materials. Because the maximum principal stress theory mentioned above is not always suitable for ductile materials, the maximum shear stress was used to evaluate the failure behavior of the TLP bonded joint. The maximum shear stress in each element can be expressed as

(5) $$\tau_{\max}=\frac{\sigma_{1}-\sigma_{3}}{2},$$

where *σ*_3_ is the third principal stress. Figure 3b1,b2 show the distribution of the maximum shear stresses in the submodel of a TLP bonded joint, where the maximum shear stress is located at the edge of the intermediate layer and at the interfaces between the intermediate layer and the parent alloy (refer to the red contour in the figure). In the intermediate layer, the maximum and average *τ*_max_ are 179.9 MPa and 155.9 MPa respectively, and the ratio between the maximum *τ*_max_ and the average *τ*_max_ is 1.15. In addition, our calculation shows that if the intermediate layer is treated as a single alloy with the same orientation as the parent alloy, no stress concentration occurs in the joint and the *τ*_max_ is 100 MPa.

To consider the influence of the second principal stress *σ*_2_ on the stress concentration of a complex stress system, the von Mises stress (equivalent stress, *σ*_eq_) was also calculated in this study. The von Mises hypothesis (i.e., the fourth strength theory) [33] holds that a material begins to yield when *σ*_eq_ reaches the yield strength and that *σ*_eq_ is given by Equation (6):

(6) $$\sigma_{\mathrm{eq}}=\sqrt{\frac{{\left(\sigma_{1}-\sigma_{2}\right)}^{2}+{\left(\sigma_{2}-\sigma_{3}\right)}^{2}+{\left(\sigma_{3}-\sigma_{1}\right)}^{2}}{2}}.$$

**Figure 4:**
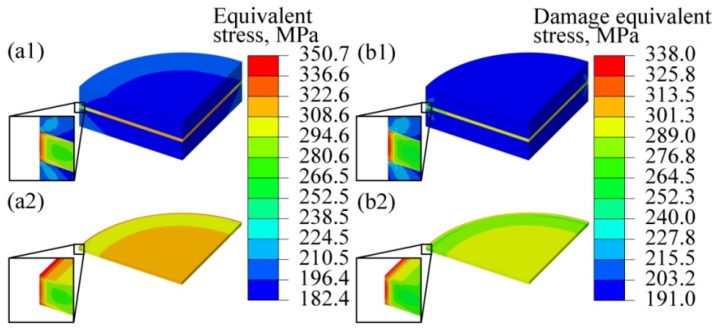
Simulation results of the von Mises equivalent stress and the damage equivalent stress: von Mises equivalent stress in the (**a1**) TLP bonded joint and (**a2**) intermediate layer, and damage equivalent stress in the (**b1**) TLP bonded joint and (**b2**) intermediate layer.

Meanwhile, to evaluate the stress triaxiality, which affects the initiation and growth of micro-voids and cracks [34] on the failure of the TLP bonded joint, the damage equivalent stress ($\sigma_{\mathrm{eq}}^{*}$) in the joint was also investigated. It can be written as follows [34]:

(7) $$\sigma_{\mathrm{eq}}^{*}=\sigma_{\mathrm{eq}}{\left[\frac{2}{3}\left(1+\nu \right)+3\left(1-2\nu \right){\left(T_{\sigma }\right)}^{2}\right]}^{\frac{1}{2}},$$

where *T_σ_* is stress triaxiality, and

(8) $$T_{\sigma }=\sigma_{m}/\sigma_{\mathrm{eq}},$$

(9) $$\sigma_{m}=(\sigma_{1}+\sigma_{2}+\sigma_{3})/3,$$

where *σ*_m_ is the hydrostatic stress. Figure 4 shows the simulation results for *σ*_eq_ and $\sigma_{\mathrm{eq}}^{*}$ in the TLP bonded joint. Obviously, both *σ*_eq_ and $\sigma_{\mathrm{eq}}^{*}$ in the intermediate layer are greater than those in the parent alloy. The *σ*_eq_ and $\sigma_{\mathrm{eq}}^{*}$ maximally distribute at the edges of the intermediate layer and at the interfaces between the intermediate layer and parent alloy (refer to the red contour in the figure), the locations of which are similar to those of the maximum *τ*_max_. The maximum *σ*_eq_ and $\sigma_{\mathrm{eq}}^{*}$ in the intermediate layer are 350.7 GPa and 338.0 GPa respectively. If the intermediate layer is treated as a single alloy with the same orientation as the parent alloy, then no stress concentration occurs and the values of *σ*_eq_ and $\sigma_{\mathrm{eq}}^{*}$ are 200 MPa (see Table S1).

**Figure 5:**
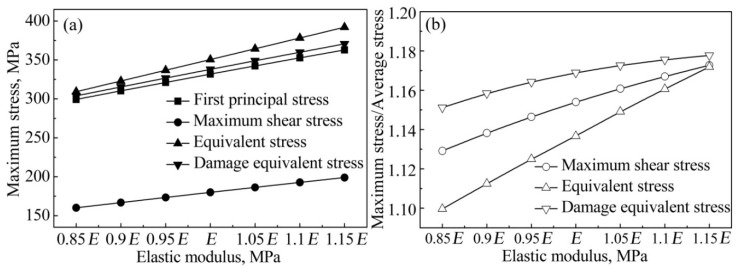
Influence of elastic moduli on (**a**) the maximum stresses and (**b**) the ratio between the maximum stress and the average stress in the TLP bonded joint.

Elastic modulus is the principal mechanical parameter for structural materials, which can be adjusted and controlled by adding alloyed elements or performing heat treatments. Figure 5 presents the influence of the elastic modulus of the intermediate layer (*E*_inter_) on the stress concentration, wherein the elastic modulus ranges from 0.85*E* to 1.15*E* (*E* = 207.378 GPa). Clearly, with the decreasing elastic modulus of the intermediate layer, the maximum stresses of first principal stress, maximum shear stress, equivalent stress, and damages equivalent stress decrease linearly. The ratio between the maximum stress and the average stress is also reduced, indicating that reducing *E*_inter_ can relieve the stress concentration. Also, the average stresses decrease linearly with decreasing elastic modulus of the intermediate layer (see Supplementary Figure S2). Therefore, in addition to increasing the mechanical strength of the intermediate layer, the simulation result in this study suggests that appropriately reducing the elastic modulus of the intermediate layer can also improve the mechanical strength of a TLP bonded joint. To verify this opinion, additional experimental work was also carried out. 

In the experiment, single-crystalline Ni_3_Al-based superalloy (named IC10 alloy) samples were prepared by TLP bonding process in a high vacuum diffusion furnace (Centorr Vacuum Industries, Workhorse II, Nashua, NH, USA) and the bonding temperature, time, and pressure were 1250 °C, 6 h, and 5 MPa respectively. The samples of TLP bonded joints and their geometry have been given in Figure 1b,c above, and the grain boundary and crystal orientation in a typical zone in the joint are presented in Supplementary Figure S3. By nanoindentation testing, as illustrated in Supplementary Figure S4, the elastic modulus and hardness of the parent alloy and intermediate-layer alloy were measured. Test results show that the average elastic modulus of parent alloy (*E*_parent_) and the average elastic modulus of intermediate-layer alloy *E*_inter_ are 221.4 GPa and 208.5 GPa respectively (see Supplementary Figure S5), and the ratio of *E*_inter_ to *E*_parent_ (i.e., *E*_inter_/*E*_parent_) is 0.942. Then, to reduce *E*_inter_ and *E*_inter_/*E*_parent_, post weld heat treatment (PWHT) was carried out in a heat treatment furnace (SG-QF1400, Shanghai, China). During PWHT, samples of TLP bonded joints were heated to about 1200°C and held for 6 h, and then cooled down in air to room temperature. Because that Boron was added to the intermediate-layer alloy as a melting-point depressant, the atomic percent of Boron in the intermediate-layer alloy was much higher than that in the parent alloy before PWHT. In the PWHT, Boron atoms diffused from the intermediate layer to the parent alloy. As the result of solution strengthening, after PWHT, *E*_parent_ increased to 223.7 GPa while *E*_inter_ decreased to 205.4 GPa, and *E*_inter_/*E*_parent_ was reduced to 0.918. According to the result of tensile testing, the average tensile strength after PWHT is 819.0 MPa at room temperature, which is higher than before PWHT (i.e., 798.0 MPa) as shown in Supplementary Figure S6. Clearly, the decrease of *E*_inter_ or *E*_inter_/*E*_parent_ can improve the mechanical strength of a TLP bonded joint, which supports the simulation result in the manuscript. Notably, the ideal strength of the TLP bonded joint should be the same as or practically be as close as possible to the strength of the parent alloy. Usually, achieving a mechanical strength of a TLP bonded joint greater than 90% of the mechanical strength of a single crystal is difficult in experiments. To further improve the mechanical strength of TLP bonded joints, sufficient attention should be devoted to the stress concentration caused by the different states of crystal orientation between the parent alloy and intermediate layer.

## 4. Conclusions

Elastic constants of single-crystalline Ni_3_Al as well as the elastic modulus, shear modulus, and the Poisson’s ratio of polycrystalline Ni_3_Al were calculated via DFT, and the calculation results were subsequently verified against previously reported experimental data. Based on the calculated mechanical properties of both single-crystalline and polycrystalline Ni_3_Al, 3D FEA was used to characterize the mechanical behavior of the TLP bonded joint of single-crystalline Ni_3_Al under a simple tension load. The simulation results revealed obvious stress concentrations in the joint as a result of different states of crystal orientation between the parent metals (single crystals) and intermediate layer (polycrystals), which will lead to failure in the polycrystalline Ni_3_Al and thereby weaken the mechanical strength of the TLP bonded joint. The maximum values of the first principal stress, maximum shear stress, equivalent stress, and the damages equivalent stress in the joint decrease linearly with decreasing elastic modulus of the intermediate layer; thus, reducing the elastic modulus of the intermediate layer can relieve the stress concentration and benefit the mechanical reliability of a TLP bonded joint, which can be verified by experiments.

## Figures and Tables

**Table 1:** Elastic constants *D_ijkl_* for single-crystalline Ni_3_Al.

Elastic Constant	*D*_1111_	*D*_1122_	*D*_1133_	*D*_2222_	*D*_2233_	*D*_3333_	*D*_1212_	*D*_1313_	*D*_2323_
Present work	240.10	160.03	160.03	240.10	160.03	240.10	123.83	123.83	123.83
Experiment [27]	224.3	148.6	148.6	224.3	148.6	224.3	125.8	125.8	125.8

**Table 2:** Elastic constants *S_ijkl_* for single-crystalline Ni_3_Al.

Elastic Constant	*S*_1111_	*S*_1122_	*S*_1133_	*S*_2222_	*S*_2233_	*S*_3333_	*S*_1212_	S_1313_	*S*_2323_
Present work	0.009	−0.004	−0.004	0.009	−0.004	0.009	0.008	0.008	0.008

## Supplementary Materials

The following are available online at https://www.mdpi.com/1996-1944/12/17/2765/s1, Figure S1: The strain conditions for deriving nine elastic stiffness coefficients; Figure S2: Relationship between the average stress and elastic modulus of intermediate layer; Figure S3: The grain boundary and crystal orientation in the TLP bonded joint: (a) SEM-EBSD image of the joint, (b) grain boundary in the joint; (c) all Euler map; and (d) IPF-Y0 map. The instrument used is FEI Quanta 650F+HKL Channel 5; Figure S4: Nanoindentation test: (a) SEM image of a typical zone in TLP bonded joint; and (b) schematic of a location of nanoindentation array (Berkovich indenter, instrument: Agilent G200); Figure S5: The influence of the post weld heat treatment (PWHT) on the elastic modulus (a) and hardness (b) of parent alloy and intermediate-layer alloy; Figure S6: The tensile strengths of TLP bonded joints before and after PWHT (three samples were measured for each point; instrument: universal testing machine, GP-TS2000M); Table S1: The stress in the joint ignoring the crystal orientation of intermediate layer.
