# Supplementary material for: Stress Concentration Induced by the Crystal Orientation in the Transient-Liquid-Phase Bonded Joint of Single-Crystalline Ni3Al
Source: Materials (Basel). 2019 Aug 28;12(17):2765. doi: 10.3390/ma12172765 (PMC6747771; doi:10.3390/ma12172765)
Supplement: Supplementary file 1 [file materials-12-02765-s001.pdf]

materials

MDPI

Supplementary Materials: Stress Concentration Induced by the Crystal Orientation in the Transient-Liquid-Phase Bonded Joint of Single-Crystalline Ni_3Al

Hongbo Qin [1,2,*], Tianfeng Kuang [1,2], Qi Li [1], Xiong Yue [1], Haitao Gao [1], Fengmei Liu [1] and Yaoyong Yi [1,*]

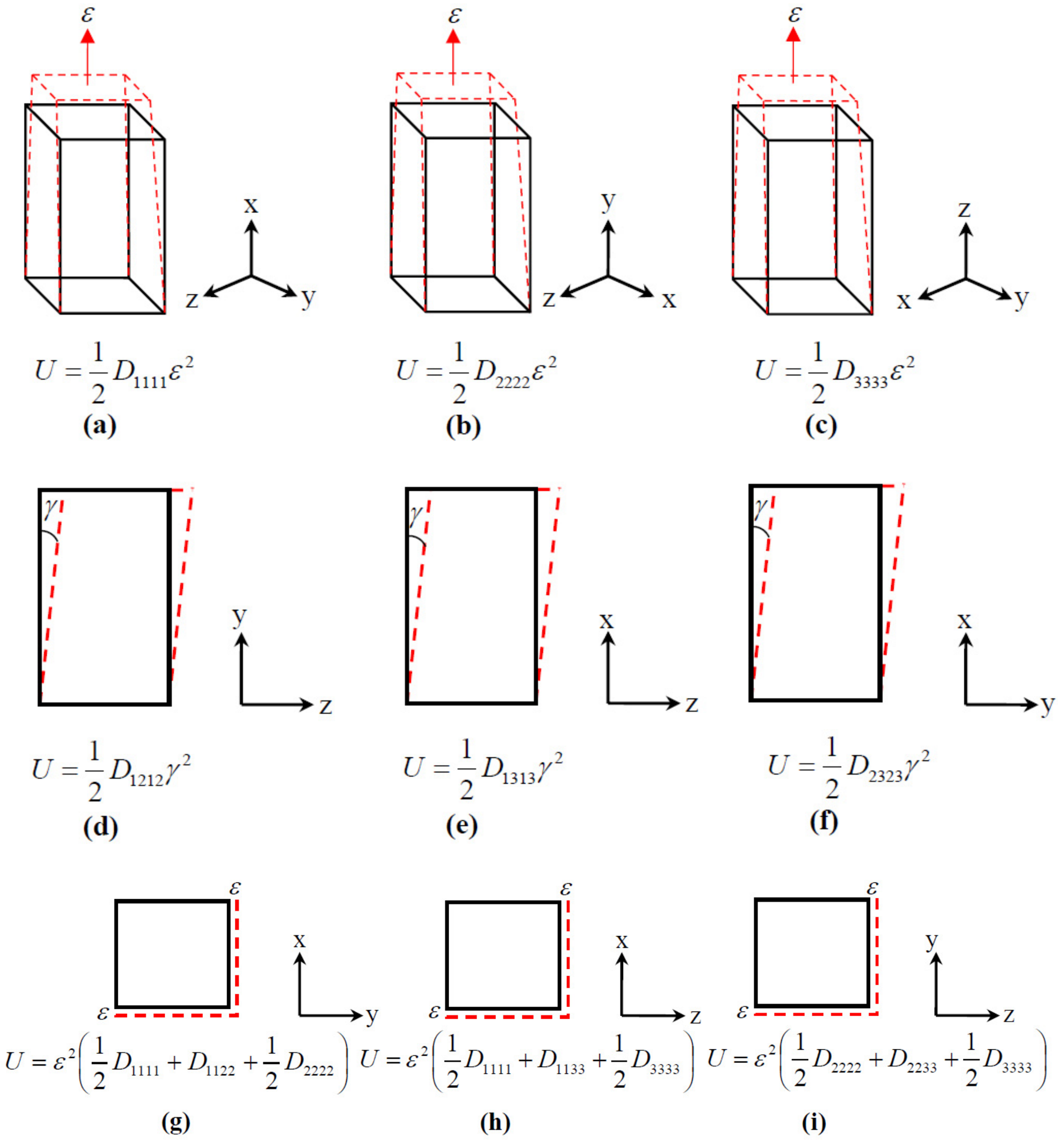

Figure S1. The strain conditions for deriving nine elastic stiffness coefficients.

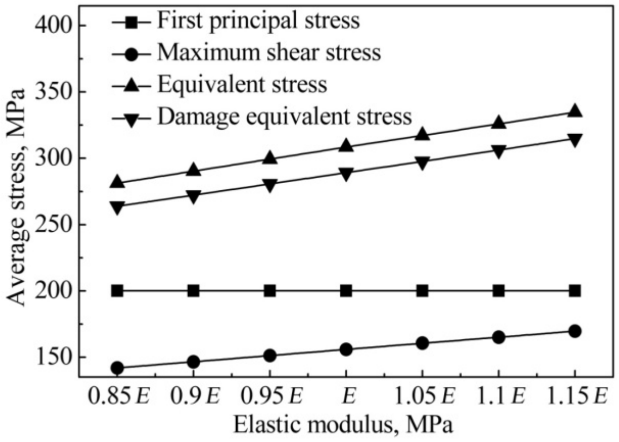

Figure S2. Relationship between the average stress and elastic modulus of intermediate layer.

Table S1. The stress in the joint ignoring the crystal orientation of intermediate layer.

**Stress**	σ_1, **MPa**	τ_{max}, **MPa**	σ_{eq}, **MPa**	σ_{eq}^{*}, **MPa**
Value	200	100	200	200

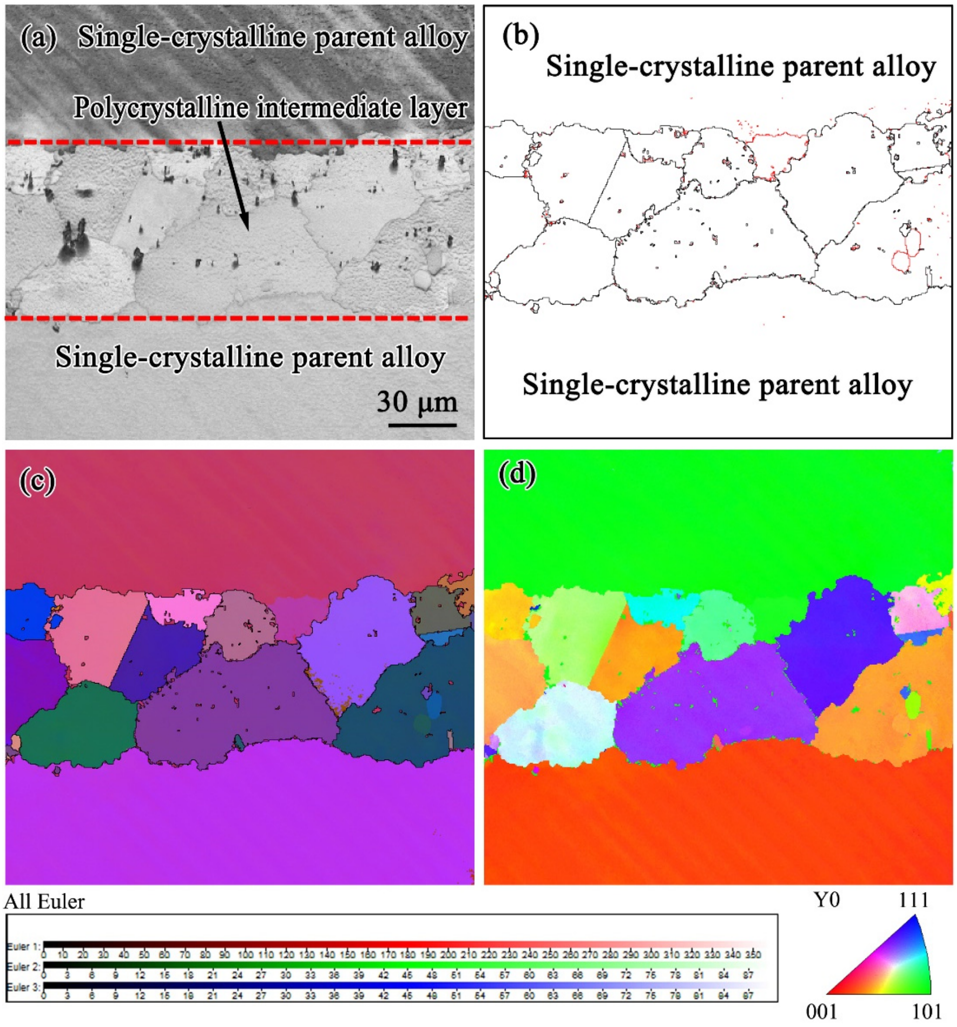

Figure S3. The grain boundary and crystal orientation in the TLP bonded joint: (**a**) SEM-EBSD image of the joint, (**b**) grain boundary in the joint; (**c**) all Euler map; and (**d**) IPF-Y0 map. The instrument used is FEI Quanta 650F + HKL Channel 5.

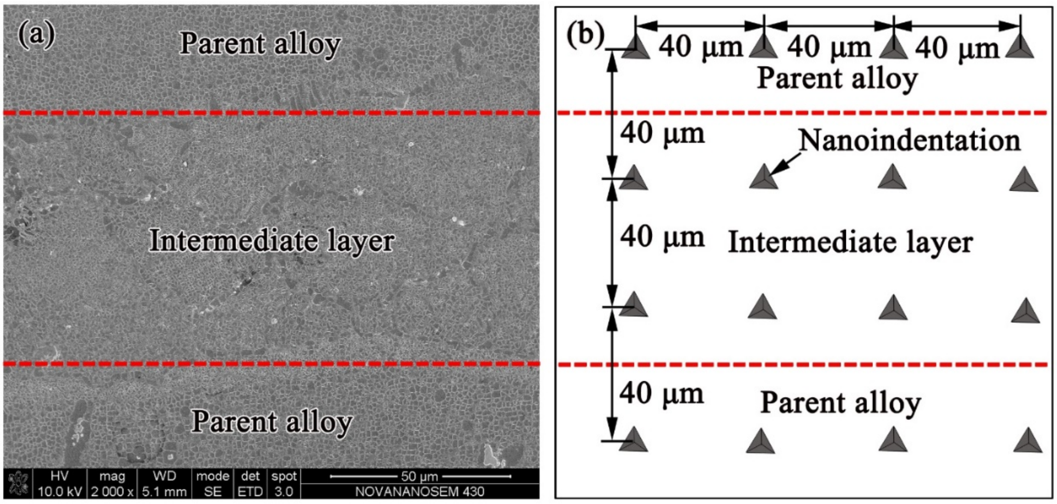

Figure S4. Nanoindentation test: (**a**) SEM image of a typical zone in TLP bonded joint; and (**b**) schematic of a location of nanoindentation array (Berkovich indenter, instrument: Agilent G200).

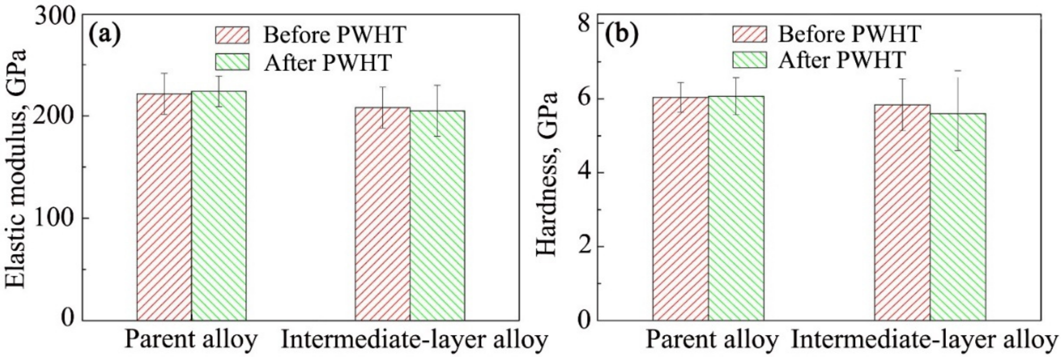

Figure S5. The influence of the post weld heat treatment (PWHT) on the elastic modulus (**a**) and hardness (**b**) of parent alloy and intermediate-layer alloy.

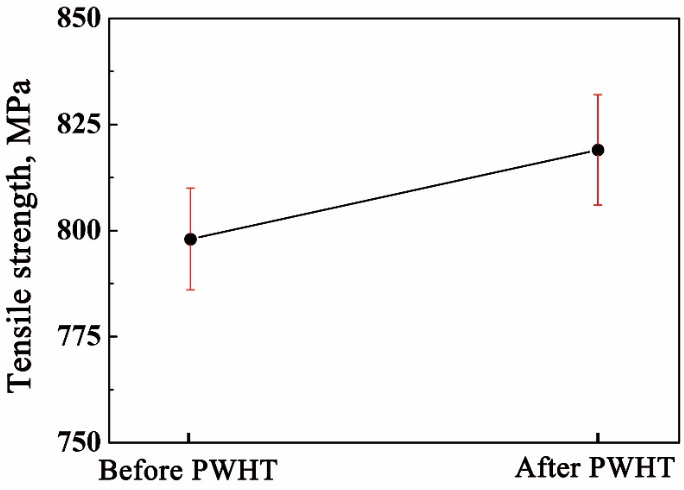

Figure S6. The tensile strengths of TLP bonded joints before and after PWHT (three samples were measured for each point; instrument: universal testing machine, GP-TS2000M).
